# Adaptive Mesh Refinement and Adaptive Time Integration for Electrical Wave Propagation on the Purkinje System

**DOI:** 10.1155/2015/137482

**Published:** 2015-10-25

**Authors:** Wenjun Ying, Craig S. Henriquez

**Affiliations:** ^1^Department of Mathematics, MOE-LSC and Institute of Natural Sciences, Shanghai Jiao Tong University, Minhang, Shanghai 200240, China; ^2^Departments of Biomedical Engineering and Computer Science, Duke University, Durham, NC 27708-0281, USA

## Abstract

A both space and time adaptive algorithm is presented for simulating electrical wave propagation in the Purkinje system of the heart. The equations governing the distribution of electric potential over the system are solved in time with the method of lines. At each timestep, by an operator splitting technique, the space-dependent but linear diffusion part and the nonlinear but space-independent reactions part in the partial differential equations are integrated separately with implicit schemes, which have better stability and allow larger timesteps than explicit ones. The linear diffusion equation on each edge of the system is spatially discretized with the continuous piecewise linear finite element method. The adaptive algorithm can automatically recognize when and where the electrical wave starts to leave or enter the computational domain due to external current/voltage stimulation, self-excitation, or local change of membrane properties. Numerical examples demonstrating efficiency and accuracy of the adaptive algorithm are presented.

## 1. Introduction

One of the long-recognized challenges in modeling cardiac dynamics [1–4] is developing efficient and accurate algorithms that can accommodate the widely varying scales in both space and time [5]. The electrical wave fronts typically occupy only a small fraction of the domain, are very sharp (in space), and change very rapidly (in time) while the electrical potential, in the region away from the wave fronts, is spatially broad and changes more slowly. With standard numerical methods on uniform grids, very small mesh parameters and very small timesteps must be used to correctly resolve the fine details of the sharp and rapidly changing wave fronts. These discretization parameters are often chosen heuristically and are fixed throughout the simulation, even if conditions change. Adaptive mesh refinement (AMR) methods have been proposed as a solution, in which coarse grids and large timesteps are used in the area where the electrical potential is changing slowly and fine grids and small timesteps are applied only in the region where the sharp electrical waves are located and the action potential changes very rapidly. Using this approach, the numbers of grid nodes and timesteps used with the adaptive algorithm are to some extent optimized. The original AMR algorithm was first proposed by Berger and Oliger for hyperbolic equations [6] and shock hydrodynamics [7]. The methods have been applied to cardiac simulations by Cherry et al. [8, 9] and Trangenstein and Kim [10].

The Berger-Oliger AMR algorithm is a hierarchical and recursive integration method for time-dependent partial differential equations. It starts time integration on a relatively coarse grid with a large timestep. The coarse grid is locally refined further if the computed solution at part of the domain is estimated to have large errors. Better solutions are obtained by continuing time integration on the fine grid with a smaller timestep until both coarse and fine grids reach the same time, called synchronization of levels. The fine grid may be locally refined further and is dynamically changing, which leads to both space and time adaptive algorithm.

The standard implementation of Berger-Oliger's AMR algorithm uses block-structured grids and assumes that the underlying grids are logically rectangular and can be mapped onto a single index space. While the method has been shown to provide computation and accuracy advantages, it is challenging to apply to domains with complex geometry.

In this paper, we present an AMR algorithm that can be used for unstructured grids. The method is applied in both idealized and realistic tree-like domains similar to that found in the His-Purkinje system. The algorithm is based on that proposed by Trangenstein and Kim [10] where operator splitting technique is used to separate the space-independent reactions part from the linear diffusion part during time integration. Both the reactions and the diffusion parts are integrated with an implicit scheme. This allows larger and adaptive timesteps. As the AMR algorithm naturally provides a hierarchy of multilevel grids, the linear systems resulting from space discretization of the linear diffusion on the adaptively refined grids are solved by a standard geometric multigrid solver. The results show that uniform coarse discretization can lead to conduction failure or changes in dynamics in some parts of the branching network when compared to a uniform fine grid. The results also show that AMR scheme with adaptive time integration (AMR-ATI) can yield results as accurate as the uniform fine grid but with a speedup of 15 times.

The remainder of the work is organized as follows.  Section 2 describes the partial differential equations, which model electrical wave propagation on the Purkinje system. Sections 3 and 4, respectively, present the time integration and space discretization for the reaction-diffusion equations. The adaptive mesh refinement and adaptive time integration procedures are outlined in Sections 5 and 6. In Section 7, some simulation results are presented with the AMR-ATI algorithm.

## 2. Differential Equations

**Figure 1:**
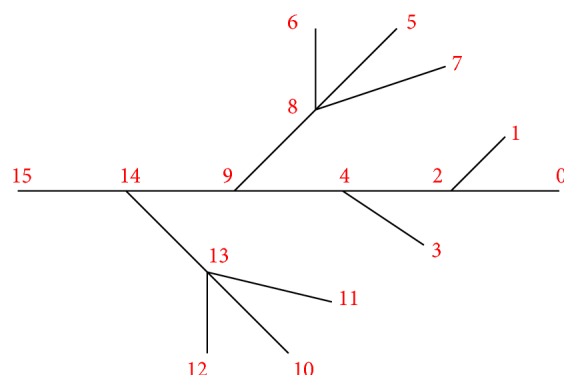
An idealized branch of the Purkinje system.

Suppose that we are given a fiber network of the Purkinje system with *N*_*V*_ vertices and *N*_*E*_ edges. See Figure 1 for an idealized branch of the Purkinje system. Let *d*_*i*_ be the number of edges connected to vertices *V*_*i*_, for each *i* = 1,2,…, *N*_*V*_. We call *d*_*i*_ the* degree* of the vertex *V*_*i*_. A vertex *V*_*i*_ with *d*_*i*_ = 1 is called a* leaf-vertex*. Otherwise, it is called a* nonleaf-vertex*. Denote the *j*th edge *E*_*j*_ in the fiber network by *E*_*j*_ = [*x*_0_^(*j*)^, *x*_1_^(*j*)^]. Denote the edges that have vertex *V*_*i*_ as the common endpoint by *E*_*i*_1__, *E*_*i*_2__,…, *E*_*i*_*d*_*i*___. Denote by *x*_*b*_*i*_1___^(*i*_1_)^, *x*_*b*_*i*_2___^(*i*_2_)^,…, *x*_*b*_*i*_*d*_*i*____^(*i*_*d*_*i*__)^ the endpoints of the adjacent edges, which overlap with vertex *V*_*i*_. The subscript *b*_*i*_*r*__ is either 0 or 1 for *r* = 1,2,…, *d*_*i*_.

On each edge *E*_*j*_ of the fiber network, the distribution of the electric/action potential is determined by the conservation of electric currents and could be described by a partial differential equation coupled with a set of ordinary differential equations. Consider

(1) a2∂J(t,x)∂x+Cm∂u(t,x)∂t+Iionu,q=Istimt,xfor  x0(j)<x<x1(j),dq(t,x)dt=M(u,q),

with

(2) $$\begin{array}{c} J\left(t,x\right)=-\frac{1}{R}\frac{\partial u\left(t,x\right)}{\partial x} \end{array}$$

as the flux. Here, *a* (units: cm) denotes a typical radius of the fiber; *C*_*m*_ (units: *μ*F/cm^2^) is the membrane capacitance constant; *R* (units: kΩ·cm) is the electrical resistivity. The vector **q** denotes a vector of dimensionless gating variables. The functions *I*_ion_(*u*, **q**) and *ℳ*(*u*, **q**) are typically nonlinear, describing the membrane dynamics of the fiber. One specific model is the Hodgkin-Huxley equations [11].

We assume the electric potential *u*(*t*, *x*) is continuous,

(3) $$\begin{array}{c} u\left(t,x_{b_{i_{1}}}^{\left(i_{1}\right)}\right)=u\left(t,x_{b_{i_{2}}}^{\left(i_{2}\right)}\right)=\cdots =u\left(t,x_{b_{i_{d_{i}}}}^{\left(i_{d_{i}}\right)}\right), \end{array}$$

and the electric flux is conserved,

(4) $$\begin{array}{c} \overset{d_{i}}{\underset{r=1}{\sum }}{\left(-1\right)}^{b_{i_{r}}}J\left(t,x_{b_{i_{r}}}^{\left(i_{r}\right)}\right)=0, \end{array}$$

at each vertex *V*_*i*_ at any time *t* > 0. At a leaf-vertex *V*_*i*_, as we have *d*_*i*_ = 1, the assumption (4) means the no-flux boundary condition is imposed.

Combined with some appropriate initial conditions for the potential function *u*(*t*, *x*) and the gating variables **q**(*t*, *x*), the differential equations above can be uniquely solved.

## 3. Time Integration and Operator Splitting

We will adapt the method of lines to temporally integrate the differential equations (1). Let *t* ^*n*^ be the discrete times, at which the equations will be discretized. At each timestep from *t* ^*n*^ to *t* ^*n*+1^, we use an operator splitting technique to advance the space-dependent part,

(5) a2∂J(t,x)∂x+Cm∂u(t,x)∂t=0 for  x0(j)<x<x1(j),

separately from the space-independent part,

(6a) $$\begin{array}{c} C_{m}\frac{\partial u\left(t,x\right)}{\partial t}+I_{\text{ion}}\left(u,q\right)=I_{\text{stim}}\left(t,x\right), \end{array}$$

(6b) $$\begin{array}{c} \frac{dq\left(t,x\right)}{dt}=M\left(u,q\right), \end{array}$$

in the differential equations (1). Note that the space-dependent part (5) is simply a linear diffusion equation. The space-independent part (6a) and (6b) is simply a set of ordinary differential equations (ODEs).

With respect to time integration, both the linear diffusion and the nonlinear reaction parts can be in principle integrated with any standard ODE solver. In this work, we adapt implicit time integration schemes to integrate both the linear diffusion equation and the nonlinear ODEs or so-called reaction equations. An implicit scheme allows relatively larger timesteps than those imposed by the stability restriction associated with an explicit scheme.

In the next section, we will focus on the space discretization of the linear diffusion equation. For simplicity, we assume the linear diffusion equation (5) is discretized in time with the backward Euler method,

(7) a2∂J(tn+1,x)∂x+Cmu(tn+1,x)−u(tn,x)Δt=0for  x0(j)<x<x1(j),

with

(8) $$\begin{array}{c} J\left(t^{n+1},x\right)=-\frac{1}{R}\frac{\partial u\left(t^{n+1},x\right)}{\partial x}. \end{array}$$

Here, Δ*t* = *t* ^*n*+1^ − *t* ^*n*^.

## 4. Space Discretization with the Finite Element Method

For conciseness, we will omit the time-dependency of the electric potential *u*(*t* ^*n*+1^, *x*) and the electric flux *J*(*t* ^*n*+1^, *x*). Let

(9) ux≡utn+1,x,  Jx≡Jtn+1,x,b(x)≡κu(tn,x)

with *κ* = 2*C*_*m*_/(*a*Δ*t*). Note that *b*(*x*) is known in the timestep from *t* ^*n*^ to *t* ^*n*+1^. The semidiscrete equation (7) can then be rewritten as

(10) −ddx1Ru′x+κux=J′x+κux=bxfor  x0(j)<x<x1(j).

In this work, we further discretize (10) with the continuous piecewise linear finite element method.

For simplicity, in this section, we only illustrate the discretization of the ODE (10) with the finite element method for the case of uniform mesh refinement.

Suppose the *j*th edge *E*_*j*_ in the fiber network is partitioned into a uniform grid, denoted by *𝒢*_*j*_. Assume the grid *𝒢*_*j*_ has (*m*_*j*_ + 1) nodes, denoted by {*ξ*_*i*_^(*j*)^}_*i*=0_^*m*_*j*_^ with *ξ*_*i*_^(*j*)^ = *x*_0_^(*j*)^ + *ih* ^(*j*)^ and *h* ^(*j*)^ = (*x*_1_^(*j*)^ − *x*_0_^(*j*)^)/*m*_*j*_.

Let *u*_*i*_^(*j*)^ be the unknown potential variable associated with the grid node *ξ*_*i*_^(*j*)^ and **u**_*h*_^(*j*)^ = (*u*_0_^(*j*)^, *u*_1_^(*j*)^,…, *u*_*m*_*j*__^(*j*)^)^*T*^ the vector of unknown potential variables. Let

(11) $$\begin{array}{c} \varphi_{i}^{\left(j\right)}\left(\xi \right)=\left\{\begin{array}{cc} \frac{\xi -\xi_{i-1}^{\left(j\right)}}{h^{\left(j\right)}} & \text{if}\;\;\xi_{i-1}^{\left(j\right)}<\xi <\xi_{i}^{\left(j\right)}\\ \frac{\xi_{i+1}^{\left(j\right)}-\xi }{h^{\left(j\right)}} & \text{if}\;\;\xi_{i}^{\left(j\right)}<\xi <\xi_{i+1}^{\left(j\right)}\\ 0 & \text{otherwise} \end{array}\right. \end{array}$$

be the continuous piecewise linear finite element basis function associated with the grid node *ξ*_*i*_^(*j*)^ for each *i* = 1,…, *m*_*j*_ − 1. At the endpoints *ξ*_0_^(*j*)^ = *x*_0_^(*j*)^ and *ξ*_*m*_*j*__^(*j*)^ = *x*_1_^(*j*)^, the associated basis functions read

(12) $$\begin{array}{c} \varphi_{0}^{\left(j\right)}\left(\xi \right)=\left\{\begin{array}{cc} \frac{\xi_{1}^{\left(j\right)}-\xi }{h^{\left(j\right)}} & \text{if}\;\;\xi_{0}^{\left(j\right)}<\xi <\xi_{1}^{\left(j\right)}\\ 0 & \text{otherwise}, \end{array}\right.\\ \varphi_{m_{j}}^{\left(j\right)}\left(\xi \right)=\left\{\begin{array}{cc} \frac{\xi -\xi_{m_{j}-1}^{\left(j\right)}}{h^{\left(j\right)}} & \text{if}\;\;\xi_{m_{j}-1}^{\left(j\right)}<\xi <\xi_{m_{j}}^{\left(j\right)}\\ 0 & \text{otherwise}. \end{array}\right. \end{array}$$

Assume the finite element solution takes the form *u*_*h*_^(*j*)^(*x*) = ∑_*i*=0_^*m*_*j*_^ *u*_*i*_^(*j*)^ *φ*_*i*_^(*j*)^(*x*), which is a linear combination of the basis functions.

We introduce the electric flux *J*(*x*) = −*R* ^−1^ *u*′(*x*) at the edge endpoints *ξ*_0_^(*j*)^ = *x*_0_^(*j*)^ and *ξ*_*m*_*j*__^(*j*)^ = *x*_1_^(*j*)^ as two extra unknowns. In terms of the basis functions {*φ*_*i*_^(*j*)^(*x*)}_*i*=0_^*m*_*j*_^, the finite element equations equivalent to the second-order ODE (10) are given by

(13) ∫011Rddxuhjxddxφijx+κuhjxφijxdx  +J(x1j)φi(x1j)−J(x0j)φi(x0j) =∫01b(x)φi(j)(x)dx

for *i* = 0,1,…, *m*_*j*_. At individual grid nodes, they explicitly read

(14) ∫ξ0(j)ξ1(j)[1Rddxuh(j)(x)ddxφ0(j)(x)+κuh(j)(x)φ0(j)(x)]dx  −Jx0jφ0jx0j=∫ξ0jξ1jbxφ0jxdx,

(15) ∫ξi−1(j)ξi+1(j)1Rddxuhjxddxφijx+κuhjxφijxdx =∫ξi−1jξi+1jbxφijxdx for  i=1,2,…,mj−1,

(16) ∫ξmj−1(j)ξmj(j)1Rddxuhjxddxφmjjx+κuhjxφmjjxdx  +J(x1(j))φmj(j)(x1(j))=∫ξmj−1(j)ξmj(j)b(x)φmj(j)(x)dx.

By further discretizing each integral in the finite element system above with the composite trapezoidal rule, we can get a set of linear equations in the following form:

(17) $$\begin{array}{c} A^{\left(j\right)}u_{h}^{\left(j\right)}+J^{\left(j\right)}=h^{\left(j\right)}b^{\left(j\right)}, \end{array}$$

with **A** ^(*j*)^ = (*a*_*r*,*s*_^(*j*)^)_(*m*_*j*_+1)×(*m*_*j*_+1)_ as the finite element stiffness matrix, **b** ^(*j*)^ = (*b*_*r*_^*j*^)_*m*_*j*_+1_ as the current vector, and **J** ^(*j*)^ as the flux vector. The stiffness matrix **A** ^(*j*)^ is tridiagonal and symmetric positive definite. In each row, at most three entries right on the diagonal (*a*_*r*,*r*−1_^(*j*)^, *a*_*r*,*r*_^(*j*)^, and *a*_*r*,*r*+1_^(*j*)^) are nonzero. The flux vector **J** ^(*j*)^ has the following form:

(18) $$\begin{array}{c} J^{\left(j\right)}={\left(-J\left(x_{0}^{\left(j\right)}\right),0,\ldots ,0,J\left(x_{1}^{\left(j\right)}\right)\right)}^{T}, \end{array}$$

where most entries are zeros except the first and the last ones.

As the fluxes *J*(*x*_0_^(*j*)^) and *J*(*x*_1_^(*j*)^) through the endpoints of each edge *E*_*j*_ are unknown, the tridiagonal system (17) involves two more unknowns than equations. So, for the global system to be uniquely solvable, we need totally 2*N*_*E*_ extra equations/conditions.

Fortunately, at a nonleaf-vertex *V*_*i*_, which has degree *d*_*i*_ > 1, we have (*d*_*i*_ − 1) equations by the continuity (3) of potentials plus one more equation by the conservation (4) of electric fluxes. At a leaf-vertex *V*_*i*_, which has *d*_*i*_ = 1, the electric flux conservation (4) provides exactly one boundary condition. Totally we have ∑_*i*=1_^*N*_*V*_^ *d*_*i*_ = 2*N*_*E*_ additional equations. The number of equations in the final system is thus the same as that of unknowns. Normally, the system is well-determined.

The overall system on the fiber network involves both the potentials at the grid nodes and the electric fluxes at the fiber vertices as unknowns. In practical computation, we eliminate the electric fluxes to get a linear system with the discrete potentials as the only unknowns. As a matter of fact, noting that *φ*_0_^(*j*)^(*x*_0_^(*j*)^) = 1 and *φ*_*m*_*j*__^(*j*)^(*x*_1_^(*j*)^) = 1, from (14) and (16), we see the electric flux *J*(*x*_0_^(*j*)^) can be simply written out in terms of *u*_0_^(*j*)^ and *u*_1_^(*j*)^ and the electric flux *J*(*x*_1_^(*j*)^) can be simply written out in terms of *u*_*m*_*j*_−1_^(*j*)^ and *u*_*m*_*j*__^(*j*)^. Plugging the explicit expressions of the electric fluxes into the conservation condition (4), we get an equation involving the unknown potentials only. After eliminating the electric fluxes, we have a well-determined system, which has *N*_*V*_ + ∑_*j*=1_^*N*_*E*_^(*m*_*j*_ − 1) equations and unknowns. It can be easily verified that the coefficient matrix of the resulting system is a strictly diagonally dominant and symmetric positive definite M-matrix [12].

In the case when the edge *E*_*j*_ is partitioned into a locally refined grid or a composite grid, the ODE (10) can be similarly discretized with the continuous piecewise linear finite element method. The coefficient matrix **A** ^(*j*)^ of the resulting system associated with each edge *E*_*j*_ is also a tridiagonal, strictly diagonally dominant, and symmetric positive definite M-matrix. The overall system of discrete equations on the fiber network is well-determined too. The electric fluxes at the fiber vertices can also be eliminated so that the resulting system has the discrete potentials as the only unknowns and the coefficient matrix is a diagonally dominant M-matrix.

In this work, the grids for the space discretization are generated by local and adaptive mesh refinement (see Section 5 for details). The discrete finite element equations on the fiber network are solved with a V-cycle multigrid method. Basic components of the V-cycle iteration on the fiber network, including presmoothing, residual restriction, coarse grid correction, correction prolongation, and postsmoothing, are essentially the same as those of the V-cycle iteration for the composite grid equations on a single interval. We refer the readers to Ying's thesis work [13] for details of the multilevel/multigrid iteration.

## 5. Adaptive Mesh Refinement

**Figure 2:**
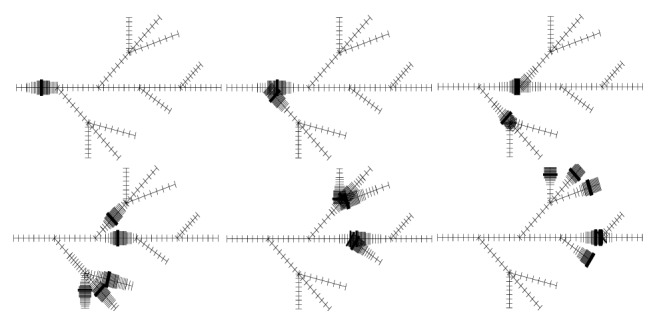
Adaptively refined grids from a simulation, which use five refinement levels.

The adaptive mesh refinement (AMR) algorithm applied for this study discretizes the fiber network into a hierarchy of dynamically, locally, and adaptively refined grids. See Figure 2 for a few composite grids, each of which consists of five locally refined grids, at different times.

The AMR algorithm follows Berger-Oliger's approach in timestepping [6–10]. It uses a multilevel approach to recursively integrate the reaction-diffusion system on the composite grids. It first integrates the system with a large timestep on a coarse level grid. Next a locally refined fine grid is generated based on available coarse data. Then the algorithm integrates the system on the fine level grid with a small timestep. Error estimation is achieved by the Richardson extrapolation [14]. By the recursive nature of the algorithm, fine grids are dynamically and locally created based on the data on coarse grids. On fine grids, smaller timesteps are used for time integration. The AMR algorithm synchronizes adjacent coarse and fine levels from time to time. At the moment of synchronization, typical routines such as mesh regridding and data up-/downscaling are performed.

Let *K* be the maximum number of mesh refinement levels and let *ℓ*_*k*_ with *k* ∈ {1,2,…, *K*} be the *k*th level of mesh refinement.  Algorithm 1 below gives a brief description of the central part of the adaptive mesh refinement procedure, which recursively advances the data at the current level *ℓ*_*k*_ and its finer levels.

**Algorithm 1 (advance(level *ℓ*_*k*_, timestep Δ*t*_*k*_)).**

*Step  1.* Integrate the differential equation at the level *ℓ*_*k*_ by its timestep Δ*t*_*k*_ with the Strang operator splitting technique.

(a) Integrate the split reaction equation by a half timestep Δ*t*_*k*_/2.
(b) Integrate the split diffusion equation by a full timestep Δ*t*_*k*_.
(c) Integrate the split reaction equation by a half timestep Δ*t*_*k*_/2.

*Step  2.* If the level *ℓ*_*k*_ has not been refined with *k* < *K* or it is time to regrid the grid on the finer level *ℓ*_*k*+1_, refine the current level *ℓ*_*k*_ or regrid the finer level *ℓ*_*k*+1_ after error estimation. Scale data down from the current level *ℓ*_*k*_ to the finer level *ℓ*_*k*+1_.

*Step  3.* Set the timestep Δ*t*_*k*+1_ = Δ*t*_*k*_/2 and integrate the finer level *ℓ*_*k*+1_ by two timesteps by recursively calling the function itself “advance(level *ℓ*_*k*+1_, timestep Δ*t*_*k*+1_).”

*Step  4.* If the finer level *ℓ*_*k*+1_ and the current level *ℓ*_*k*_ are synchronized, reaching the same time, scale data up from the finer level *ℓ*_*k*+1_ to the current level *ℓ*_*k*_.

**Figure 3:**
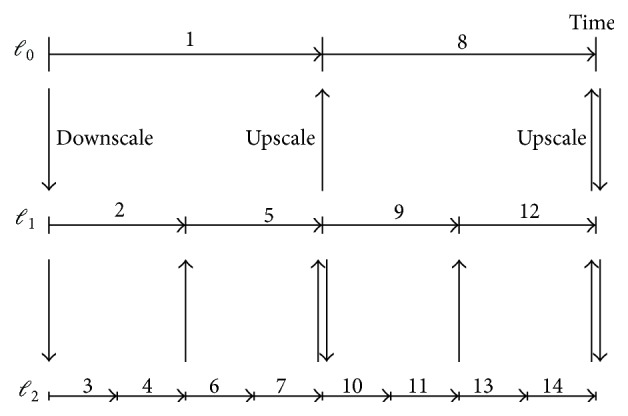
Recursive advancing of three mesh refinement levels.

Figure 3 illustrates the recursive advancing or integration of three different mesh refinement levels. Coarse levels are advanced/integrated before fine levels. Each level has its own timestep of different size: a coarser level has a larger timestep size and a finer level has a smaller timestep. The algorithm first advances level *ℓ*_0_ by Δ*t*_0_ (indicated by “1”), next advances level *ℓ*_1_ by Δ*t*_1_ = Δ*t*_0_/2 (indicated by “2”), and then advances level *ℓ*_2_ by two steps with Δ*t*_2_ = Δ*t*_1_/2 (indicated by “3” and “4”). Upon the synchronization of levels *ℓ*_1_ and *ℓ*_2_, data on the fine level *ℓ*_2_ are upscaled to the coarser level *ℓ*_1_. The recursive integration continues with Steps “5,” “6,” “7,” and so forth.

The design of the AMR algorithm used in this work was based on a few assumptions proposed by Trangenstein and Kim [10].

- First we assume the number of elements in the base grid, which describes the computational domain and is provided by the user and is relatively small. This will allow linear systems on the grid to be solved very quickly in the middle of a multilevel (composite grid) iteration and will also improve the performance of the adaptive algorithm. The base grid is fixed during the process of adaptive mesh refinement. Other grids on fine levels are in general dynamically and recursively generated by local or uniform refinement of those on coarse levels.
- Second, we assume that the region covered by the grid on a fine level is contained in the interior of that on the coarser level unless both coarse and fine grids coincide with physical boundary of the computational domain. This assumption can prevent recursive searching for element neighbors. This assumption is called* proper level nesting*.
- Third, we assume that if an element of a coarse grid is refined in any part of its physical space, it must be refined everywhere. As a result, the boundary of the main grid on a fine level aligns with the boundary of a subgrid of that on the previous coarser level. By a subgrid of a grid, we mean the union of a subset of its elements. This assumption is called* alignment of level grids*.
- Fourth, after the data on a coarse level grid are advanced by some time, we assume that the data on its finer level are advanced by as several timesteps as required by stability and accuracy to reach exactly the same time as the coarse level. This assumption implies that a coarse level is integrated before its finer levels and the timestep on a coarse level is an integer multiple of that on the next finer level. It also implies that the time stepping algorithm must be applied recursively within each timestep on all but the finest level. This assumption is called* synchronization of advancement*.
- Fifth, by the time a fine level and its coarser level are synchronized, we assume that data on the fine level is more accurate than that on the coarse level. So, the data on a fine level must be upscaled to its coarser level before the coarse level is further advanced by another coarse timestep. This assumption is called* fine data preference*.
- Sixth, as stated, all grids except the coarsest one in the AMR algorithm are changing dynamically. It is necessary for a coarse level to regrid its finer level from time to time. But it will be extremely costly to tag elements and regrid levels every coarse timestep. So, we would rather make regridding infrequently. At a coarse level, the number of timesteps between times of regridding its finer level is called* regrid interval*. In the AMR algorithm, the regrid interval may be chosen to be an integer divisor of the refinement ratio [13], which could be any even integer number in the implementation. This assumption is called* infrequent regridding*.
- Finally, there is one more assumption on the adaptive algorithm, called* finite termination*. This means that the user shall specify a maximum number of refinement levels. Once the refinement reaches the maximum level, no further mesh refinement is performed.

As determined by the nature of the Purkinje system, the grids resulting from space discretization of the computational domain are essentially unstructured. This restricts direct application of Berger-Oliger's original AMR algorithm, which requires the underlying grid to be Cartesian or logically rectangular. The AMR algorithm adapted here for the Purkinje system works with unstructured grids. It represents an unstructured grid by lists of edges and nodes. A grid node is allowed to have multiple connected edges and the degree of a node could be greater than two. The unstructured grid representation can naturally describe the Purkinje system, which primarily is a tree-like structure but has some loops inside.

Finally, it is worth mentioning that in the AMR algorithm, there is a critical parameter, called AMR tolerance and denoted by tol_AMR_, which is used by the Richardson extrapolation process for tagging coarse grid elements. The AMR tolerance closely influences efficiency and accuracy of the algorithm. The larger the tolerance is, the more efficient the algorithm is but the less accurate the solution is. The smaller the tolerance is, the more accurate the solution is but the less efficient the algorithm is. Due to the limitation of space, the detailed explanation of the Richardson extrapolation, the AMR tolerance, and other components of the AMR algorithm, such as tagging and buffering of coarse grid elements, implementation of the V-cycle multigrid iteration on the adaptively refined grids, and the data up-/downscaling between coarse and fine grids, are all omitted. We refer interested readers to Ying's dissertation [13].

## 6. Adaptive Time Integration

The AMR algorithm can automatically recognize when and where the wave fronts start to leave or enter the computational domain due to external current/voltage stimulation or self-excitation. Once the algorithm finds that the wave fronts have left the computational domain and determines that there is no need to make any further mesh refinement, it will turn it off and work with adaptive time integration (ATI) only. The implementation of timestep size control for the adaptive time integration is standard. In each timestep, the ODEs/reactions are integrated with a full timestep once and independently integrated with a half timestep twice. Then the two solutions are compared and an estimation of relative numerical error is obtained by the standard Richardson extrapolation technique [13]. If the estimated (maximum) relative error, denoted by ‖*E*‖_max⁡_, is greater than a maximum relative tolerance rtol_ATI_^(max⁡)^, the timestep size was rejected and a new timestep will be estimated by

(19) $$\begin{array}{c} \Delta t^{\left(\text{new}\right)}=\gamma \cdot {\left(\frac{\text{rto}{\text{l}}_{\text{ATI}}^{\left(\max\right)}}{{\left\|E\right\|}_{\max}}\right)}^{1/(p+1)}\cdot \Delta t^{\left(\text{old}\right)}, \end{array}$$

and the Richardson extrapolation process is repeated. Otherwise, the solution with two half timesteps is simply accepted (actually an extrapolated solution could be used for better accuracy). Here, the coefficient *γ* = 0.8 is called a safety factor, which ensures that the new timestep size will be strictly smaller than the old one to avoid repeated rejection of timesteps. The constant *p* in the exponent of (19) is the accuracy order of the overall scheme.

If the suggested timestep size is less than a threshold timestep, which usually indicates that there is an abrupt/quick change of solution states, a local change of membrane properties or arrival of an external stimulus, the adaptive mesh refinement process, is then automatically turned back on again. In the implementation, the threshold timestep is not explicitly specified by the user. It is set as the timestep that is used on the base grid.

In addition, during the ATI period, if the estimated relative error ‖*E*‖_max⁡_ is less than a minimum relative tolerance rtol_ATI_^(min⁡)^, a slightly larger timestep,

(20) $$\begin{array}{c} \Delta t^{\left(\text{new}\right)}=1.1\Delta t^{\left(\text{old}\right)}, \end{array}$$

is suggested for integration in the next step. The adaptive time integration does not like timestep size to be significantly increased within two consecutive steps for the implicit solver to have a good initial guess. For the same reason, a maximum timestep size Δ*t* ^(max⁡)^ is imposed during the adaptive time integration. This usually guarantees that the Newton solver used converges within a few iterations.

## 7. Results

The AMR algorithm proposed in the work was implemented in custom codes written in C++. The simulations presented in this section were all performed in double precision on a dual Xeon 3.6 GHz computer. No data was output when the programs were run for timing studies.

In all of the numerical studies, the electrical resistivity *R* and the membrane capacitance *C*_*m*_ are fixed to be constant, 0.5 kΩ·cm and 1 *μ*F/cm^2^, respectively. The membrane dynamics are described by the Beeler-Reuter model [15]. No-flux (homogeneous Neumann-type) boundary conditions were applied at the leaf-nodes of the fiber network.

During the time integration for the time-dependent PDEs, a second-order operator splitting technique, the Strang splitting, is applied in each timestep. The ODEs resulting from operator splitting are integrated with a second-order singly diagonally implicit Runge-Kutta (SDIRK2) scheme. The linear diffusion equation is temporally integrated with the Crank-Nicolson scheme and spatially discretized with the continuous piecewise linear finite element method. The finite element system on locally refined grids is solved with a V-cycle multigrid/multilevel iterative method in each timestep at each refinement level. (In the V-cycle composite grid iteration, the multigrid prolongation is done by the piecewise linear interpolation. The multigrid restriction is implemented in a way so that the restriction matrix is the transpose of the prolongation matrix. At the coarsest level, as the coefficient matrix of the discrete system is a symmetric and positive definite M-matrix, the linear equations are solved with a symmetric successive overrelaxation preconditioned conjugate gradient method.) The resulting scheme has second-order global accuracy if the tolerances in each component of the adaptive algorithm are consistently selected [13].

In the adaptive simulations, the mesh refinement ratio is fixed to be two and the critical AMR tolerance tol_AMR_ is set as 0.02. The maximum and minimum relative tolerances, which are used in the ATI period, are chosen to be rtol_ATI_^(max⁡)^ = 10^−2^ and rtol_ATI_^(min⁡)^ = 10^−4^, respectively. We restrict the timestep sizes used in the adaptive time integration so that they are not greater than one millisecond, that is, Δ*t* ^(max⁡)^ = 1 msec. The value of *p* in the exponent of (19) is equal to two as the scheme has second-order accuracy.

**Example 2 .**

Simulations on a two-dimensional branch with thickness/radius-variable and nonuniform branch segments. Voltage stimulation is applied from right.

**Figure 4:**
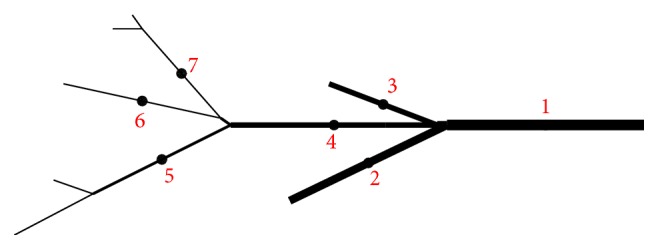
A typical branch in the heart conduction system is highly nonuniform in the sense that the lengths of branch segments, each of which is bounded by two adjacent leaves or branching vertices, may vary significantly. In this figure, branch segments “1” and “4” are not directly connected. Instead, they are connected through a very short branch segment. Similarly, branch segments “4” and “6” are also connected through a very short segment.

The two-dimensional branch shown in Figure 4 has a dimension of 4 cm × 2 cm, which is bounded by the rectangular domain [0,4]×[1,3] cm^2^. This two-dimensional branch has variable (piecewise constant) radius. Branch segments “6” and “7” have the smallest radius, equal to 20 *μ*m. Branch segment “1” has the largest radius, equal to 160 *μ*m and eight times that of branch segment “6.” The radius of branch segment “2” is six times that of “6.” Branch segments “3” and “4” have the same radius, four times that of “6.” The radius of branch segment “5” is twice that of branch segment “6.”

A voltage stimulation is applied from the right end of branch segment “1” through a discontinuous initial action potential, which is given as follows:

(21) $$\begin{array}{c} V_{m}\left(x,y\right)=\left\{\begin{array}{cc} 25.4\;\text{mvolts} & \text{if}\;\;x>3.5\\ -84.6\;\text{mvolts} & \text{otherwise}. \end{array}\right. \end{array}$$

The base grid used by the simulation with adaptive mesh refinement is a coarse partition of the branch structure (Figure 4) and has 240 edges with minimum element size equal to 0.0078 cm and maximum element size equal to 0.078 cm. Note that this is a highly nonuniform grid as the ratio of the maximum to the minimum element size is much greater than one. Actually, in the simulation, the base grid was generated by three times uniform bisection from a much coarser grid, which only has 30 edges. This makes the multigrid iterations for linear systems more efficient even though its contribution to the speedup of the overall algorithm is marginal as most (>90%) of the computer time used by the simulation was spent integrating nonlinear ODEs/reactions. The adaptive simulation effectively uses three refinement levels. To apply the Richardson extrapolation technique, the second level grid was created from uniform bisection of the base grid. In fact, only the grids at the third level were dynamically changing. They are regridded every other timestep.

In the adaptive simulation, the timestep sizes vary from Δ*t* = 0.0098 msecs to Δ*t* = 1.0 msec. The AMR process was automatically turned off at time *t* = 42.85 msecs as the algorithm determines there is no need to make spatially local refinement. After that, the ATI process is activated. The simulation on the time interval [0,400] msecs totally used 99.1 secs in computer time.

The two-dimensional branch is also partitioned into a fine, quasi-uniform grid, which has 726 edges with minimum element size equal to 0.0104 cm and maximum element size equal to 0.0127 cm. The simulation with this fine, quasi-uniform grid and timestep Δ*t* = 0.00635 msecs on the same time interval as the adaptive one used about 1406.0 secs in computer time. We call this one “uniform” simulation.

Another simulation simply working with the coarse grid, which is used as the base grid for the AMR one, is also performed. We call this as “no-refinement” simulation. The timestep is fixed to be Δ*t* = 0.039 msecs. This one used 76.7 secs in computer time.

**Figure 5:**
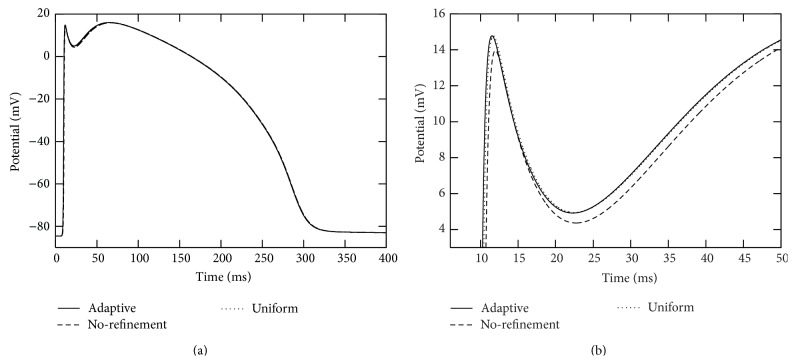
Traces of action potentials during the simulation period [0,400] msecs at marked point “4” in the two-dimensional branch shown in Figure 4. The solid curves were from the adaptive simulation. The dotted curves were from the uniform simulation. The dashed curves were from the “no-refinement” simulation. In these simulations, a voltage stimulation is applied from the right side of the radius-variable branch structure. The right plot is a close-up of the left plot.

**Figure 6:**
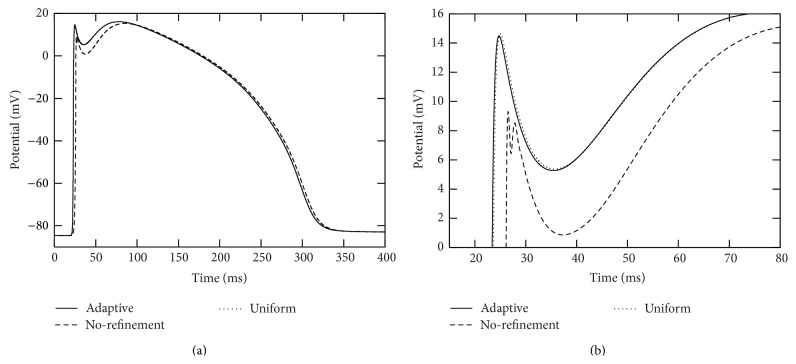
Traces of action potentials during the simulation period [0,400] msecs at the point marked as “7” in the two-dimensional branch shown in Figure 4. The solid curves were from the adaptive simulation. The dotted curves were from the uniform simulation. The dashed curves were from the “no-refinement” simulation. In these simulations, a voltage stimulation is applied from the right side of the thickness/radius-variable branch structure. The right plot is a close-up of the left plot.

In each of the simulations above, the action potential is initiated at the right end of branch segment “1” and propagates across the whole computational domain. We collected action potentials at those seven marked points (see Figure 4) during the simulations. See Figures 5 and 6 for traces of action potentials at the marked points “4” and “7,” respectively. The action potentials from the adaptive and uniform mesh refinement simulations match very well, and their difference at the peak of the upstroke phase is uniformly bounded by 0.1 mV. The action potential from the “no-refinement” simulation has least accurate results. Potential oscillation is even observed at point “7” (see Figure 6) in the “no-refinement” simulation.

**Example 3 .**

Simulations on a two-dimensional branch whose segments have piecewise constant radii. Voltage stimulation is applied from top.

We once again run three simulations, respectively, with adaptive, uniform mesh refinement and no-refinement. The computational domain and parameters are all the same as those used previously. The only difference now is to stimulate the tissue from top instead of from right.

The voltage stimulation is applied from the upper part of branch “7” through a discontinuous initial action potential, which is given as follows:

(22) $$\begin{array}{c} V_{m}\left(x,y\right)=\left\{\begin{array}{cc} 25.4\;\text{mvolts} & \text{if}\;\;y>2.8\\ -84.6\;\text{mvolts} & \text{otherwise}. \end{array}\right. \end{array}$$

**Figure 7:**
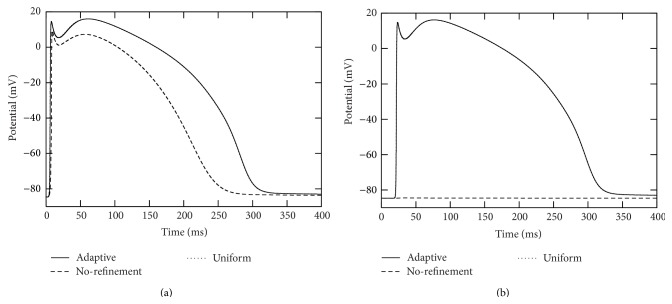
Traces of action potentials during the simulation period [0,400] msecs at marked points “7” and “6” in the two-dimensional branch shown in Figure 4. The solid curves were from the adaptive simulation. The dotted curves were from the uniform simulation. The dashed curves were from the “no-refinement” simulation. In these simulations, a voltage stimulation is applied from the top of the radius-variable branch structure. The left plot corresponds to the marked point “7” and the right plot corresponds to the marked point “6.”

The action potential successfully propagates across the whole computational domain in each of the adaptive and uniform refinement simulations while it fails to pass across branching points where the fiber radius changes from small to large in the “no-refinement” simulation (see Figure 7).

To verify accuracy of the solution from the adaptive simulation, action potentials at the seven marked points (see Figure 4) are also collected and compared with those from the simulation with the fine and quasi-uniform grid. It is observed that the adaptive and uniform results match very well too, and their difference at the peak of the upstroke phase is bounded by 0.15 mvolts.

The simulation with adaptive mesh refinement used 100.4 msecs in computer time. The timestep sizes used in the adaptive simulation vary from Δ*t* = 0.0098 msecs to Δ*t* = 1.0 msec. The adaptive mesh refinement was automatically turned off at time *t* = 44.02 msecs.

The one with the uniform grid and timestep Δ*t* = 0.00635 msecs used 1405.0 msecs in computer time.

**Example 4 .**

Simulations on a three-dimensional branch whose segments have piecewise constant radii.

**Figure 8:**
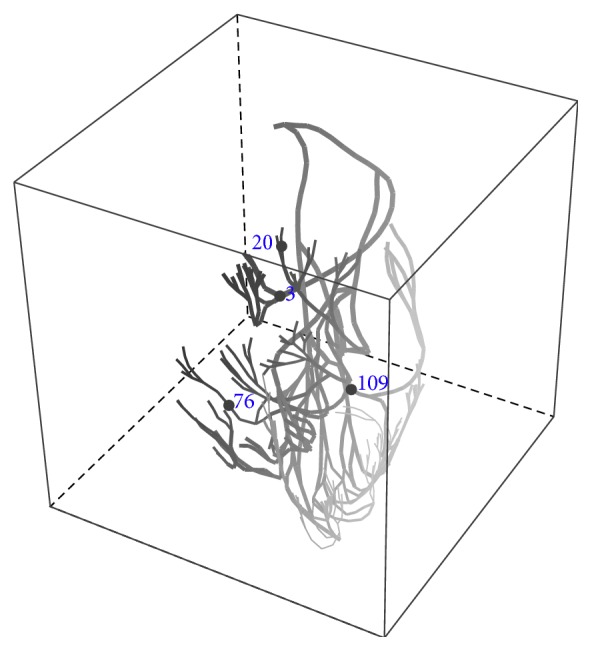
The idealized Purkinje system of the heart.

The three-dimensional Purkinje system used in the following simulations was originally from 3Dscience.com. The fiber radius of the system was modified to be piecewise constant for simplicity. The radius of each branch segment takes one of the five values as the two-dimensional case. The minimum and the maximum radius of the fiber are, respectively, 20 *μ*m and 160 *μ*m. Intermediate values are 40, 80, and 120 cm. See Figure 8 for an illustration of the topological structure, where the ratios of variable radii are not courteously shown.

The idealized Purkinje system has a dimension 2.04 × 2.16 × 2.98 cm^3^. It is bounded by the rectangular domain, [−0.39,1.65]×[4.05,6.21]×[−1.31,1.67] cm^3^. The maximum of the shortest paths from the top-most vertex to other leaf-vertices, computed by Dijkstra's algorithm is about 5.29 cm.

As in the two-dimensional examples, in each of the simulations with the idealized Purkinje system, a voltage stimulation is applied from the top branch through a discontinuous initial action potential, given as follows:

(23) $$\begin{array}{c} V_{m}\left(x,y,z\right)=\left\{\begin{array}{cc} 25.4\;\text{mvolts} & \text{if}\;\;z>1.372\\ -84.6\;\text{mvolts} & \text{otherwise}. \end{array}\right. \end{array}$$

The base grid used by the adaptive simulation is a coarse partition of the Purkinje system and has 1412 edges/elements with minimum element size equal to 0.006 cm and maximum element size equal to 0.075 cm. This is also a highly nonuniform grid as the ratio of the maximum to the minimum element size is up to 12.5. Once again, in this simulation, the base grid was actually generated from uniform bisection of a coarser grid, which has 706 edges/elements as this will make the multigrid iterations for linear systems more efficient. The adaptive simulation effectively uses three refinement levels. Similar to the two-dimensional case, the second level grid was created from uniform bisection of the base grid and only the grids at the third level were dynamically changing, regridded every other timestep.

The timestep sizes used in the adaptive simulation vary from 0.00935 msecs to 1.0 msec. The adaptive mesh refinement was automatically turned off at time *t* = 42.74 msecs. The simulation on the time interval [0,400] msecs totally used 626.7 secs in computer time.

The three-dimensional Purkinje system is also partitioned into a fine and quasi-uniform grid, which has 6311 edges/elements with minimum element size equal to 0.0095 cm and maximum element size equal to 0.016 cm. The simulation with this fine quasi-uniform grid and timestep Δ*t* = 0.0082 msecs used about 9696.0 secs in computer time.

We also run a simulation with timestep Δ*t* = 0.0374 msecs on the coarse grid that is used as the base grid for the adaptive simulation. This “no-refinement” simulation used 458.0 secs in computer time.

**Figure 9:**
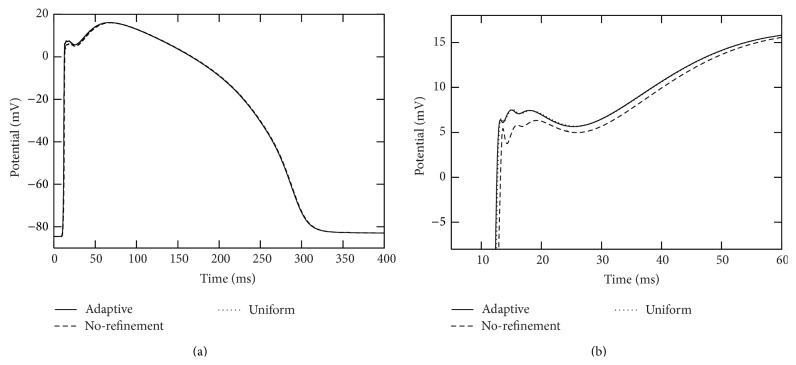
Traces of action potentials during the simulation period [0,400] msecs at the point marked as “3” in Figure 8. The right plot is a close-up of the left one.

**Figure 10:**
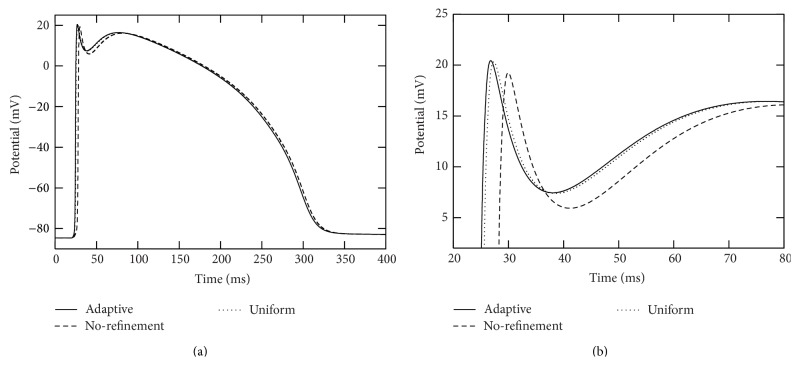
Traces of action potentials during the simulation period [0,400] msecs at the point marked as “20” in Figure 8. The right plot is a close-up of the left one.

**Figure 11:**
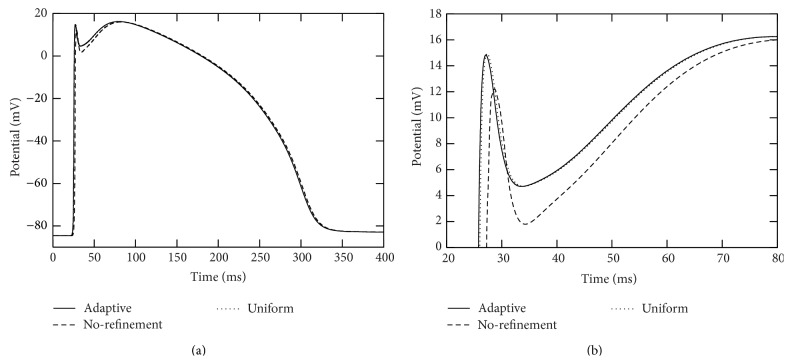
Traces of action potentials during the simulation period [0,400] msecs at the point marked as “76” in Figure 8. The right plot is a close-up of the left one.

**Figure 12:**
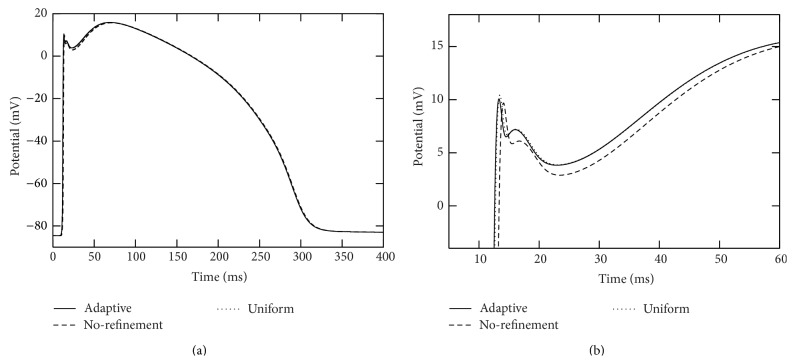
Traces of action potentials during the simulation period [0,400] msecs at the point marked as “109” in Figure 8. The right plot is a close-up of the left one.

**Figure 13:**
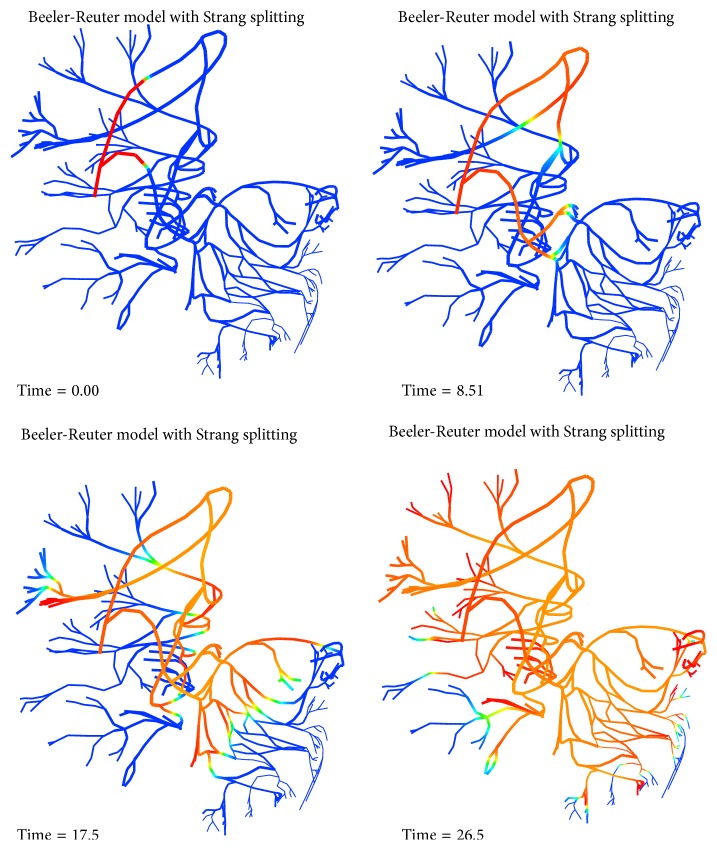
Four plots of the action potential at different times by the AMR-ATI simulation (red denotes activated potential and blue denotes inactivated potential).

It is observed that, in each of the adaptive, uniform refinement and no-refinement simulations, the action potential successfully propagates and goes through the whole domain. The solution from the adaptive simulation is in very good agreement with that from the uniform simulation. We collected action potentials at sixteen points located in different regions of the domain and found that the corresponding potential traces almost overlap. Their difference at the peak of the upstroke phases is uniformly bounded by 0.4 mvolts. See Figures 9, 10, 11, and 12 for some visualized results, which correspond to action potentials at the four marked points shown in Figure 8.  Figure 13 shows four plots of the action potential at different times by the AMR-ATI simulation. The adaptive simulation roughly uses the same computer time as the “no-refinement” one but yields much more accurate results and gains about 15.5 times speedup over the uniform one.

## 8. Discussion

This paper demonstrates the promising application of a both space and time adaptive algorithm for simulating wave propagation on the His-Purkinje system.

In this work, only the numerical results with membrane dynamics described by Beeler-Reuter model are presented. The adaptive algorithm, however, is by no means restricted to the simple model. In fact, in our implementation, the algorithm successfully works with other physically realistic models, such as Luo-Rudy dynamic model and DiFrancesco-Noble model. As the advantage of the algorithm due to application of the operator splitting technique, principally any standard cardiac model of reaction-diffusion type for modeling wave propagation can be easily incorporated into the adaptive algorithm.

In addition, the adaptive algorithm can also be straightforwardly applied to modeling signal propagation on the neural system of the body or even the brain.

Other than its advantages, admittedly the algorithm has its own limitations. For example, efficiency of the adaptive algorithm heavily relies on the existence of a relatively coarse base grid, which describes the computational domain. As the speedup of an adaptive mesh refinement algorithm is roughly bounded by the ratio of the maximum degree of freedoms (DOFs) of the grid with the highest resolution to the mean DOFs of all grids that have ever been created during the adaptive process, the existence of a coarse base grid makes the ratio as large as possible and hence its speedup over a simulation on the grid with highest resolution. The use of a relatively coarse base grid also makes the multigrid/multilevel solver for linear systems more efficient. It is noticeable that in our simulations the base grids were all selected to have the number of edges/elements as small as possible.

In the AMR algorithm [10, 13] designed for uniform or quasi-uniform grids, the timestep size is typically selected to be proportional to the minimum of element sizes (multiplied by an estimated wave speed) in a grid in order that the numerical waves or discontinuities will never propagate more than one element within a single timestep. As this strategy guarantees that the fronts of traveling waves are always bounded and tracked by fine grids, the ODEs resulting from operator splitting applied to the reaction-diffusion systems are only integrated on the fine grids while the solution in other regions covered by coarser level grids is simply obtained by time interpolation.

However, a typical branch in the heart conduction system or the whole system on its own is highly nonuniform in the sense that the lengths of branch segments, each of which is bounded by two adjacent leaves or branching vertices, may vary significantly (see Figure 4), which determines that the coarse partition of the domain is also highly nonuniform since the algorithm requires the number of edges/elements as small as possible for the efficiency considerations. On this kind of computational domain, the determination of timesteps based on minimum element sizes is found to be less efficient. Here, in the proposed adaptive algorithm, timestep sizes are instead chosen to be proportional to the maximum of the element sizes in a grid. This alternative strategy is experimentally proved to be efficient and accurate enough in simulating electric waves that travel moderately fast even though its rigidity needs further investigation, in particular when the fiber radius is much larger than those currently used.

In the simulations presented in this work, error estimations in both the AMR and ATI processes were all based on the Richardson extrapolation process, which is in general more expensive but more rigorous and reliable than other simpler ones such as the gradient detector. If the gradient detector is used, the second coarsest grid in the AMR process does not need to be uniformly bisected and can also be changing dynamically, and the ATI period can use less computer time. The potential gain in algorithm efficiency and the possible loss in solution accuracy with the alternative use of a gradient detector can be studied and compared in a future work.

Finally, we make a remark about the parallelization of the space-time adaptive mesh refinement and adaptive time integration algorithm on the Purkinje system. Our numerical experiments in this work indicate that a large fraction (about 80%) of the computer time in the simulation is spent in the time integration for the reaction part while the V-cycle multigrid iteration as well as the mesh refinement part uses only a small fraction (less than 20%). The dominance of the computer time in the split reaction part is partially due to the one-dimensional nature of the Purkinje network system (mesh refinement and grid generation on one-dimensional structures are much easier than the ones with multiple dimensions). It is obvious that this dominance will be beneficial for the parallelization of the algorithm since the split reaction part (ODEs) is spatially independent, highly parallelizable, and can even be massively parallelized. Of course, the time consumption on different parts of the operator splitting depends on the specific cardiac models. With a more complicated cardiac model, the reaction part will consume more computer time than the diffusion part and the corresponding parallel algorithm is expected to have better efficiency. Admittedly, with the reaction part fully parallelized, further improvement in parallel efficiency will depend on the parallelization of the V-cycle multigrid iteration, which solves linear systems over the whole fiber network and needs time in data communication between different CPUs or multicores. The parallel performance of the AMR-ATI algorithm deserves further investigation.
